# Confounding Factors to Predict the Awakening Effect-Site Concentration of Propofol in Target-Controlled Infusion Based on Propofol and Fentanyl Anesthesia

**DOI:** 10.1371/journal.pone.0124343

**Published:** 2015-05-04

**Authors:** Shun-Ming Chan, Meei-Shyuan Lee, Chueng-He Lu, Chen-Hwan Cherng, Yuan-Shiou Huang, Chun-Chang Yeh, Chan-Yang Kuo, Zhi-Fu Wu

**Affiliations:** 1 Department of Anesthesiology, Tri-Service General Hospital and National Defense Medical Center, Taipei, Taiwan, Republic of China; 2 School of Public Health, National Defense Medical Center, Taipei, Taiwan, Republic of China; 3 Graduate Institute of Medical Sciences, National Defense Medical Center, Taipei, Taiwan, Republic of China; University of Bari, ITALY

## Abstract

We conducted a large retrospective study to investigate the confounding factors that predict Ce ROC under propofol-based TIVA with TCI. We recorded sex, age, height, weight, Ce LOC, Ce ROC, total propofol and fentanyl consumption dose, and anesthetic time. Simple linear regression models were used to identify potential predictors of Ce ROC, and multiple linear regression models were used to identify the confounding predictors of Ce ROC. We found that Ce ROC correlated with age, sex, Ce LOC, and both total fentanyl and propofol consumption dose. The prediction formula was: Ce ROC = 0.87 - 0.06 × age + 0.18 × Ce LOC + 0.04 (if fentanyl consumption > 150 μg; if not, ignore this value) + 0.07 × (1 or 2, according to the total propofol consumption dose, 1 for a propofol amount 1000-2000 mg and 2 for a propofol amount > 2000 mg). We simplified the formula further as Ce ROC = 0.87 - 0.06 × age + 0.18 × Ce LOC. In conclusion, Ce ROC can be predicted under TCI with propofol- and fentanyl-based TIVA. The confounding factors that predicted propofol Ce ROC are age, sex, Ce LOC, and total consumption dose of propofol and fentanyl.

## Introduction

The target-controlled infusion (TCI) machine provides a function to estimate the effect-site concentration (Ce) and the elimination time of propofol. Iwakiri and Nishihara (2005) suggested that patients can be expected to awaken quickly upon completion of the procedure and discontinuation of drug administration if we know the propofol effect-site concentration at loss of consciousness (Ce_LOC_) [1]. Nunes and Ferreira (2005) reported that the propofol effect-site concentration at return of consciousness (Ce_ROC_) was related to Ce_LOC_ and the patients age, and they concluded that Ce_ROC_ can be estimated by combining information about Ce_LOC_ and the patients age [2]. Shafer and Doze (1998) found that Ce_ROC_ was related to the patients age, sex, weight, and type of surgery [3]. By knowing the associated factors that predict Ce_ROC_, the anesthesiologist should be able to estimate the emergence time and to provide a fast emergence to shorten the anesthesia-controlled time. However, the three reports mentioned above included small sample sizes. The purpose of this large retrospective study was to estimate the confounding factors that predicted propofol Ce_ROC_ under TCI.

## Materials and Methods

This retrospective study retrieved information from the electronic database and anesthetic records of the Tri-Service General Hospital (TSGH; Taipei, Taiwan, Republic of China). The Ethics Committee of TSGH approved the study (TSGHIRB No: 100-05-168). IRB allow waiving the requirement for obtaining informed consent and patient records was anonymized and de-identified prior to analysis. Data collected by six different anesthesia providers with average twelve years of experience. At least two anesthesiologists independently reviewed the case information in the database and the medical records for that case. The study included 794 patients (464 women and 330 men) classified as American Society of Anesthesiologists (ASA) physical status I to III, who were aged 18–88 years and were scheduled for one of the following types of surgery: elective surgery of the extremities; spine surgery; exploratory laparotomy; laparoscopy; ear, nose, or throat surgery; or ophthalmological, genitourinary, chest, gynecological or breast cancer surgery. The exclusion criteria were known neurological disorders, pregnancy, medication affecting the central nervous system, uncontrolled hypertension, recent use of psychotropic drugs, chronic alcohol consumption, severe obesity (body mass index > 35 kg m^2−1^), combined inhalation anesthesia with propofol, or incomplete data.

### Anesthetic techniques used in our routine practice

No medication was administered before induction of anesthesia, as in our clinical practice. Regular monitoring included electrocardiography (lead II), pulse oximetry, and noninvasive measurement of blood pressure, respiratory rate, and end-tidal carbon dioxide pressure. Three electrodes [A-Line Auditory Evoked Potential electrodes; Danmeter, Odense, Denmark] were positioned at the mid-forehead (+), left forehead (reference), and left mastoid (–). Anesthesia was induced using a total intravenous anesthesia (TIVA) technique with intravenous (i.v.) fentanyl (2–3 μg kg^−1^) and 2% lidocaine (1.5 mg kg^−1^). Continuous infusion of propofol (Fresfol 1%) was delivered using a TCI system (Base Primea, Fresenius Kabi AG, Bad Homburg, Germany) with Schnider’s kinetic model of a Ce of 3.0–5.0 μg ml^−1^. Propofol was adjusted to keep the Auditory Evoked Potential Index (AAI) between 15 and 25 during maintenance of anesthesia.

Rocuronium (10 mg, i.v.) was given as required by the return of neuromuscular function. We maintained the hemodynamic parameters in the 20% range of preoperative values. Hypertension and tachycardia were treated with 1 μg kg^−1^ of i.v. fentanyl if the AAI was in the set range. After two unsuccessful treatments, 5 mg of i.v. labetalol was given. Hypotension was treated with fluid, and 5 mg of i.v. ephedrine was given if the AAI was in the set range. Atropine (0.5 mg) was given if the HR was < 50 bpm and accompanied by hypotension [4]. TCI of propofol was turned off at the last surgical suture. At the end of the surgical procedure, 2 mg neostigmine and 1 mg atropine were given intravenously. The conduct of anesthesia, including fluid management, was determined by the attending anesthesiologist. When the patient regained consciousness with smooth respiration, the tracheal tube was removed and the patient was sent to the post-anesthesia recovery room for further care.

In our anesthesia chart and computer system, we had recorded age, sex, height, weight, Ce_LOC_, Ce_ROC_, anesthesia time, surgical time, and both propofol and fentanyl consumption dose. Anesthesia time was defined as the time from anesthesia induction to extubation of the tracheal tube, and surgical time was defined as the time from the skin incision to covering with the dressing. Emergence time was defined as the time from the end of surgery to extubation. The total dose of propofol and fentanyl were defined as the total dose of propofol and fentanyl administered from the induction of general anesthesia to cessation of drug administration. Ce_LOC_ was defined as the propofol effect-site concentration at the time of loss of consciousness without eyelash reflex, and Ce_ROC_ was defined as the propofol effect-site concentration at the time when the patient opened his/her eyes in response to his/her name being called loudly at 30-s intervals, which corresponds with Observer’s Assessment of Alertness/Sedation Score 4–5.

### Statistical analysis

The patients’ characteristics are presented as minimum, maximum, mean, and standard deviation. Before multivariable analysis, data are needed thorough univariate analyses between independent variables and dependent variable, such as correlation analysis.Simple linear regression models were used to identify potential predictors of Ce_ROC_. Multiple linear regression models were used to assess the independent predictors for Ce_ROC_. Statistical analyses were performed using SPSS Version 16.0 software (SPSS Inc, Chicago, III). Statistical significance was defined as *p*< 0.05.

## Results

The patients’ characteristics are shown in Table 1. According to the records, anesthesia induction and maintenance were smooth in all cases. No patient reported memory of the operation either spontaneously or when questioned about it on the day after the operation.

**Table 1:** Patients’ characteristics (330 men, 464 women).

	Minimum	Maximum	Mean (SD)
Age (yr)	18	88	48.6 (16.4)
Height (cm)	138	194	163 (8.53)
Weight (kg)	37	120	63.4 (12.2)
BMI (kg m^2 −1^)	14.5	34.7	23.9 (3.72)
Total propofol (mg)	337	8170	1254 (809)
Propofol (mg kg^−1^ min^−1^)	0.041	0.204	0.120 (0.025)
Fentanyl (μg kg^−1^ min^−1^)	100	1000	209 (99.5)
Ce_LOC_	1.30	5.00	2.94 (0.483)
Ce_ROC_	0.500	1.80	1.17 (0.263)
Surgical time (min)	40	820	150 (108)
Anesthetic time (min)	50	897	176 (118)

Anesthetic time = induction + maintain + emergence time

**Fig 1:**
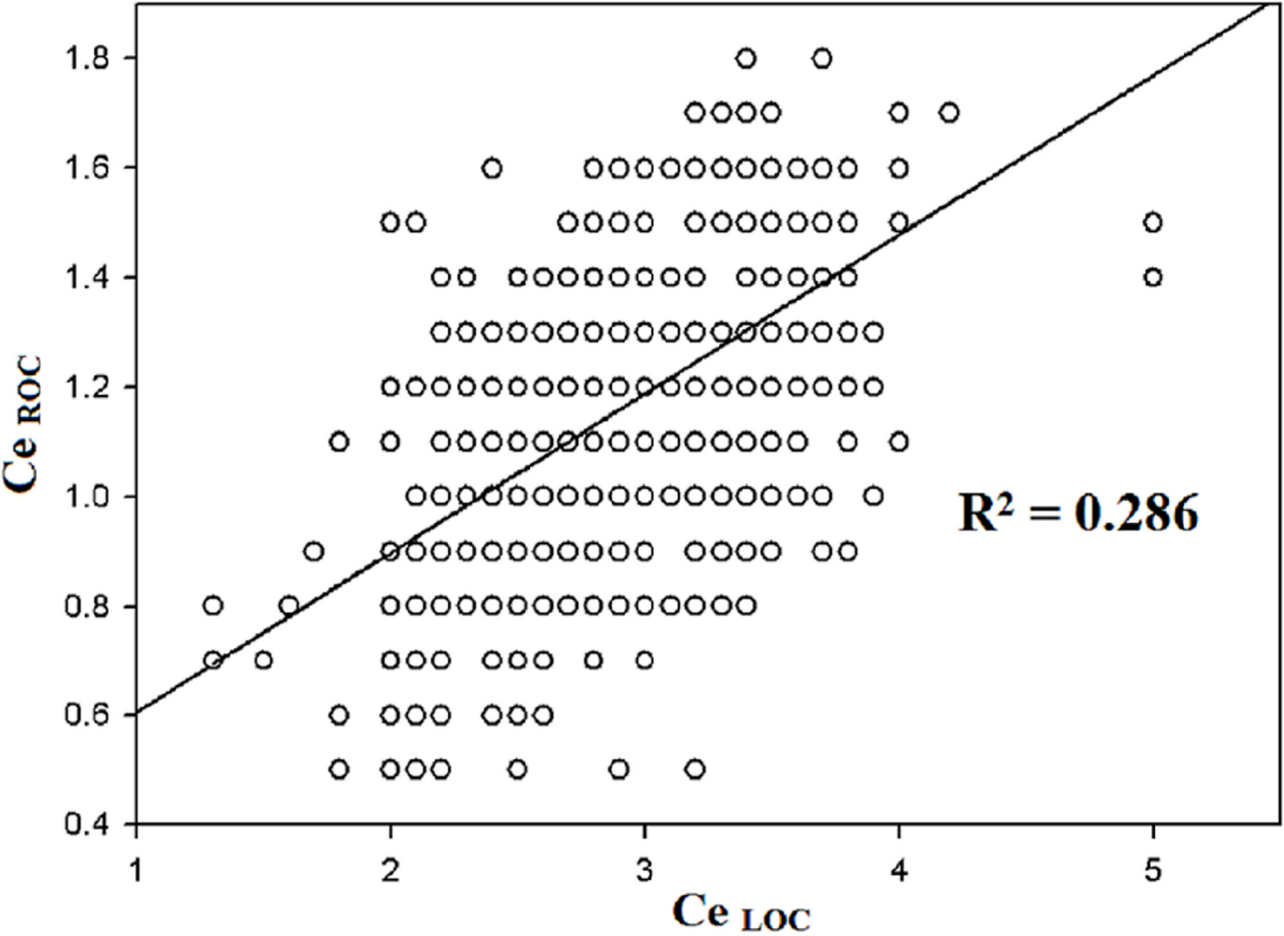
Linear regression between Ce_ROC_ and Ce_LOC_ in 794 patients. Statistically significant correlation (R^2^ = 0.286; *p* < 0.01) and positive slope.

**Fig 2:**
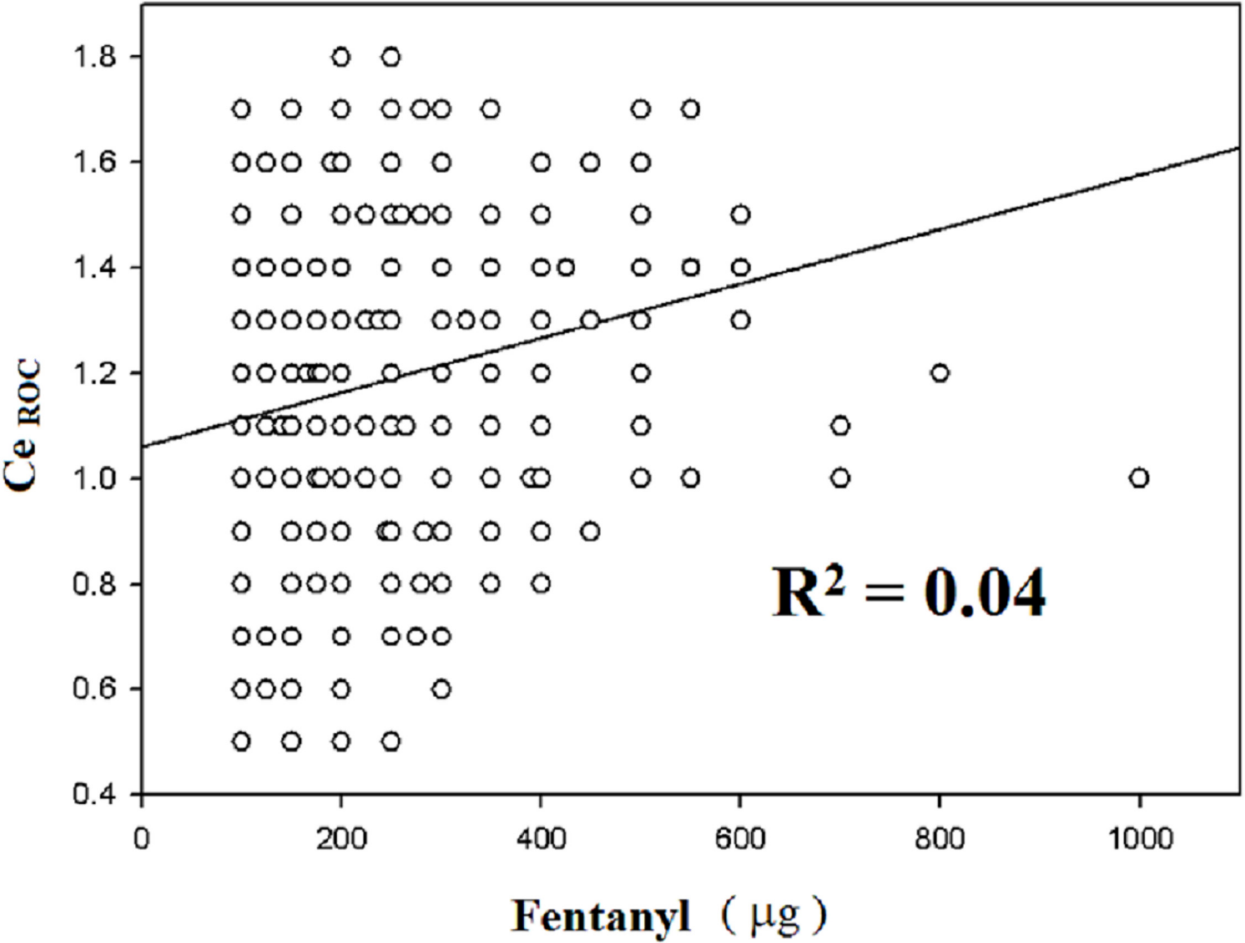
Linear regression between Ce_ROC_ and consumption of fentanyl in 794 patients. Statistically significant correlation (R^2^ = 0.04; *p* < 0.01) and positive slope.

**Fig 3:**
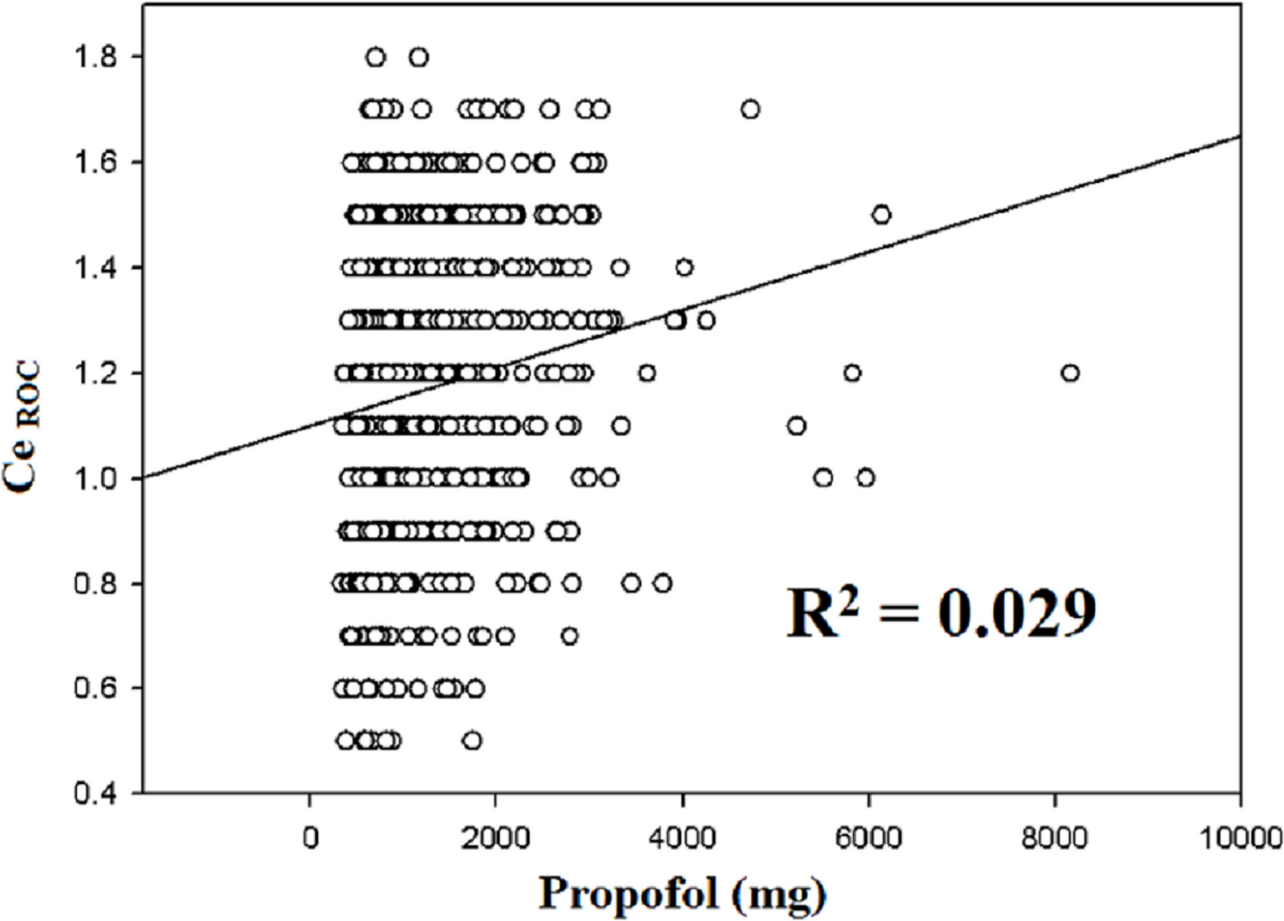
Linear regression between Ce_ROC_ and consumption of propofol in 794 patients. Statistically significant correlation (R^2^ = 0.029; *p* < 0.01) and positive slope.

**Fig 4:**
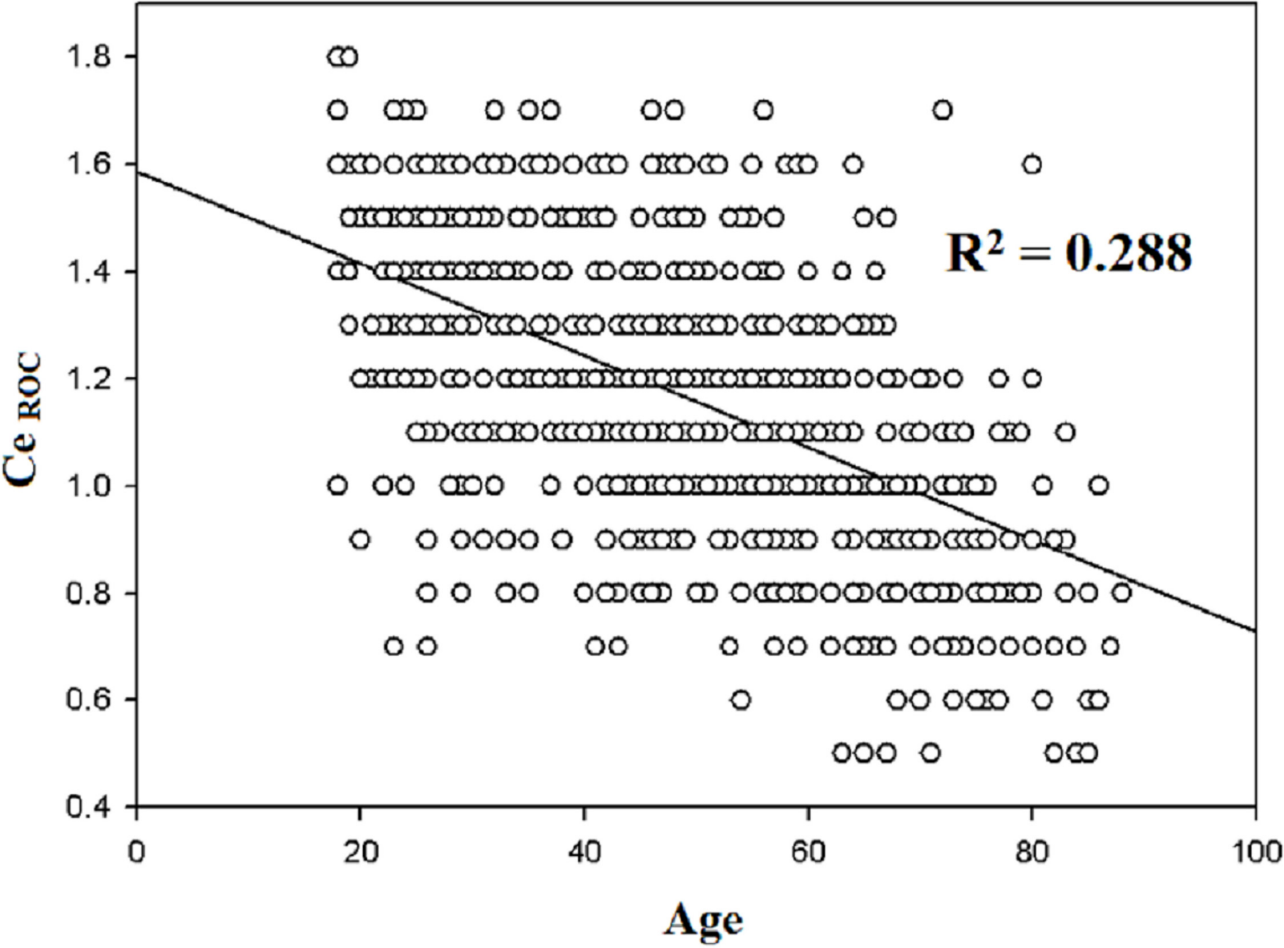
Linear regression between the Ce_ROC_ and age in 794 patients. Statistically significant correlation (R^2^ = 0.288; *p* < 0.01) and negative slope.

The regression coefficient (β) and coefficient of determination (R^2^) against Ce_ROC_ are shown in Table 2. In the univariate analyses, positive correlations were observed between Ce_ROC_ and Ce_LOC_ (R^2^ = 0.286, *p* < 0.01, Fig 1), between Ce_ROC_ and fentanyl consumption (R^2^ = 0.04, *p* < 0.01, Fig 2), and between Ce_ROC_ and propofol consumption (R^2^ = 0.029, *p* < 0.01, Fig 3). A negative correlation was observed between age and Ce_ROC_ (R^2^ = 0.288, *p* < 0.01, Fig 4). The formula from a multiple linear regression analysis was obtained as:

**Table 2:** Regression coefficient (β) and coefficient of determination (R^2^) against the return of consciousness of effective concentration of propofol (Ce_ROC_) (n = 794).

	Simple linear regression model				Multiple linear regression model			
	β	SE	R^2^	p-value	β	SE	R^2^	p-value
							.432	<.001
Ce_LOC_	.291	.016	.286	<.001	.181	.017		<.001
Gender (ref: F)	.073	0.19	.019	<.001				
Age, 10 yr	-.086	.005	.288	<.001	-.061	.005		<.001
BMI	-.003	.002	.001	.287				
Anesthetic time (hr)	.004	.005	.001	.346				
Surgical time (hr)	.005	.005	.001	.376				
Fentanyl (μg) (ref: ≤ 150)			.040	<.001				
>150	.107	.019			.042	.016		.008
Propofol consumption (mg) (ref: <1000)			.029	<.001				
1000–2000	.057	.020			.073	.016		<.001
>2000	.130	.028			.146	.023		<.001
Constant					.867	.067		<.001

$$\begin{array}{l} Ce_{ROC}=0.867-0.061\times \;"\text{age}"\;\left("\text{every}\;10\;\text{yrs}"\right)+0.181\times \;Ce_{LOC}+0.042\;\times \;\left(\text{fentanyl}>150\;\mu \text{g}\right)\\ +0.073\;\left(\text{propofol}>\;1000-2000\;\text{mg}\right)+0.146\;\left(\text{propofol}>\;2000\;\text{mg}\right)\;\left(R^{2}=0.432\right). \end{array}$$

This equation could be simplified as the following prediction formula (round off to the 2nd decimal place):

$$\begin{array}{l} Ce_{ROC}=0.867-0.06\;\times \;"\text{age}"\;\left("\text{every}\;10\;\text{yrs}"\right)+0.18\;\times \;Ce_{LOC}+0.04\\ \times \;\left(\text{fentanyl}>\;150\;\mu \text{g};\text{if not},\text{ignore this value}\right)+0.07\times "1"\;\text{or}\;"2"\;(1\;\text{or}\;2,\text{according to}\\ \text{total propofol consumption amount},\;1\;\text{for propofol}\;1000-2000\;\text{mg and}\;2\;\text{for amount}>\;2000\;\text{mg}). \end{array}$$

Both Ce_LOC_ (3.01 ± 0.48 vs. 2.88 ± 0.48 μg ml^−1^ respectively, *p* < 0.001) and Ce_ROC_ (1.21 ± 0.29 vs. 1.13 ± 0.23 μg ml^−1^ respectively, *p* < 0.001) were higher in men than in women. Consumption of both propofol (0.111 ± 0.024 vs. 0.127 ± 0.024 mg kg^−1^ min^−1^ respectively, *p* < 0.001) and fentanyl (0.021 ± 0.010 vs. 0.025 ± 0.012 μg kg^−1^ respectively, *p*< 0.001) was lower in men than in women.

## Discussion

The major finding of our study is that Ce_ROC_ correlated with age, sex, Ce_LOC_, and consumption dose of both total fentanyl and propofol. These results are consistent with those of previous studies [1, 3, 5]. The two key factors that predicted Ce_ROC_ were age and Ce_LOC_. We simplified the formula further as *Ce*_*ROC*_ = 0.87 − 0.06 × age × 0.18 × *Ce*_*LOC*_. (Because variables in fentanyl and propofol are very small to affect outcome).This result is consistent with the study by Nunes, which reported the equation *Ce*_*ROC*_ = 0.8215 + 3.0589 × *Ce*_*LOC*_/age [5]. If we apply our Ce_LOC_ and age data to the formula of Nunes, we obtain a Ce_ROC_ of 1.01 and about 10% variation to produce a Ce_ROC_ of 1.17 with our formula. Thus, these two formulas may be able to predict Ce_ROC_. This study provides a new strategy to address problem about predicting the awakening effect-site concentration.

We found that the patients’ age correlated inversely with Ce_ROC_ in patients undergoing TIVA under TCI with propofol. That is, the older the patient, the lower the propofol Ce_ROC_. This finding corroborates the results of Nunes, who found a significant correlation between Ce_ROC_ and patients’ age when using a different induction technique in 31 patients [5]. They reported that older patients had a lower Ce_ROC_ with the same Ce_LOC_. This may be explained by the fact that the weight of the human brain decreases by about 10% with age; the gray matter decreases more than the white matter [6]. Kreuer and Schreiber (2005) also found that the average normalised propofol consumption decreases with increasing patients age [7]. Schnider and Minto (1998) reported an increased sensitivity to propofol’s effect in elderly patients [8]. These data suggest that propofol dose should be reduced in elderly patients for pharmacokinetic and pharmacodynamic reasons [8, 9]. Schnider created a model of the age-related pharmacodynamic relationship between plasma propofol concentration and loss of consciousness, which we used in the present study.

We found that Ce_LOC_ correlated positively with Ce_ROC_ in patients undergoing TIVA under TCI with fentanyl. This finding is also consistent with the results of previous studies [1, 5]. Iwakiri concluded that propofol Ce_ROC_ can be estimated using Ce_LOC_ with the Marsh model and that Ce_LOC_ can be used as a guide to anaesthetic management to maintain a constant concentration [1]. They also theorized that such a clinical management strategy could be better than simply targeting the population-based average effect-site concentration. Nunes also reported that Ce _ROC_ is positively related to Ce_LOC_ [5].

We also found that sex was significantly related to Ce _ROC_ in patients undergoing TIVA under TCI with fentanyl. These results are consistent with previous studies showing that females emerge faster from TIVA than males [3, 10, 11]. The reason may reflect a possible hormonal influence on the effect of hypnotic drugs. In our study, both propofol and fentanyl consumption was higher in women than in men, possibly because females have a larger proportion of body fat and smaller water content compared with males [12]. This would affect the volume of distribution and therefore the initial concentration of many drugs used in anesthesia [12]. For lipid-soluble drugs, such as opioids and benzodiazepines, the volume of distribution is generally larger in females [12]. Conversely, for water-soluble drugs, such as neuromuscular blocking agents, the volume of distribution is generally smaller in females. The pharmacokinetic analysis produced a larger volume of redistribution and higher clearance in women, which may explain why females consume more propofol than males [13]. However, sex was not linearly related in the multiple linear regression analysis; possibly because of the inclusion of sex as nominal data.

We found that fentanyl and propofol consumption correlated positively with Ce _ROC_ in patients undergoing TIVA under TCI. Analgesic and hypnotic drugs may interact with each other to achieve an adequate depth of anesthesia during surgery [12]. The analgesic concentration of opioid can affect the predictive performance of TCI [14–16]. We found that patients with more propofol consumption presented higher Ce _ROC_ and the result might conflict with our knowledge, as we know the context-sensitive half-time increases after longer infusion times [17]. However, patients sometimes underwent prolonged anesthesia. Although the propofol consumption increased, the TCI machine provided a fixed Ce and then decreased the infusion rate with time. Moreover, the maintained propofol was guided by AAI, and we observed that the maintained Ce decreased with time. In addition, we used fentanyl mainly in the first 2 h of the operation, after which its administration was dependent on each patient’s hemodynamics. Therefore, the fentanyl dose must have been < 1.5 ng ml^−1^ in prolonged surgery (> 3 h). At a lower level of analgesia, the patient returns to consciousness at a higher hypnotic level, which may explain why the longer infusion in our study had a higher Ce _ROC_ [18]. Thus, our formula for predicting Ce _ROC_ produced a higher propofol and fentanyl consumption and Ce _ROC_. Although the results were statistically significant, the clinical effect was minimal.

Operating room (OR) time is expensive, and it is estimated that the cost of one OR to the health consumer or insurance carrier is about US $10–30 per minute [19]. Surgeons, anesthesiologists, and hospital administrators continue to try to find ways to increase OR efficiency and profitability in the face of decreasing insurance coverage and increasing costs of health care. In clinical practice, the ability to predict revenue and clinical productivity is important for strategic planning for anesthesiology groups to reduce total procedure time, induction and emergence from anesthesia. Moreover, if the total reduction in non-operative time is large enough to allow more patients to be treated in the OR during regular business hours, the overall financial impact must be considered. Recently, we included data from 1405 patients, with 595 patients receiving TIVA and 810 receiving desflurane anesthesia in ophthalmic surgery. The extubation time was faster (1.85 min) and the PACU stay time was shorter (3.62 min) in the TIVA group than in the desflurane anesthesia group [20]. In our previous studies, TIVA using a TCI system provided faster emergence than volatile anesthesia in different surgeries, which increased the OR turnover rate [18, 21–22]. These results will have an economic impact on increasing OR productivity and reducing labor costs because our ORs are consistently used for more than 8 hours daily. Chiang and Wu (2013) demonstrated that TCI systems may facilitate the clinical management of TIVA in endoscope examination [23]. The anesthesiologist sets only the desired effect-site concentration as the target and the TCI pump adjusts the rate of delivery of the anesthetic agent according to a pharmacokinetic model. It is also important for evaluating the profitability of providing care to the general TIVA under TCI procedure. Previous studies have reported that these TCI anesthetic techniques are associated with a high level of patient and surgeon satisfaction, faster recovery time, and better hemodynamic and respiratory stability and safety [23–25].

Previous studies have reported conflicting results. Vuyk reported that the propofol concentrations at which 50% and 90% of the patients showed loss of consciousness were 3.4 μg ml^−1^ and 4.34 μg ml^−1^, respectively [26]. We found a lower Ce_LOC_ than that reported by Vuyk, possibly because their study did not include opioid administration [26]. Shafer reported that the predicted blood propofol concentrations at which 50% and 95% of patients were awake after surgery were 1.07 μg ml^−1^ and 0.52 μg ml^−1^, respectively, with intermittent bolus propofol [3]. In our study, we calculated the Ce of propofol and delivered propofol with the TCI machine. Schuttler and Kloos (1988) reported a Ce _ROC_ of 1.59 μg ml^−1^ after 131 min of TCI propofol infusion, and Jung and Yang (2011) reported a Ce _ROC_ of 1.4 μg ml^−1^ and Ce_LOC_ of 3.4 μg ml^−1^ with the Schnider model [27–28]. The values in these reports are all higher than those in our study. The differences between our study and the previous studies may relate to differences in the ethnicity of the patients, shorter propofol infusion times compared with ours (176 min), and the use of different opioids (sufentanil vs. fentanyl).

There are some limitations to this study. First, it was a retrospective study. (For example: without analgesic protocol) Second, our data reflect the experiences of a single academic medical center and therefore may be biased toward the hospital’s subspecialization. Third, the study lacks systematically collected data about patient satisfaction and potential adverse sequelae of the technique involved. Fourth, propofol causes hypotension, particularly in volume depleted patients [29]. In this study, transient hypotension was resolved by fluid challenge and ephedrine treatment from anesthetic records. This may be due to this study excluded emergent surgery and less volume depleted patients. Fifth, the retrospective feature of the study does not allow to analyze important data such as impact of propofol on cerebral hemodynamics and the effect of propofol on postoperative recall [30–31]. Finally, because the R^2^ was only 0.432 for the formula presented here, it is likely that more confounding factors are involved, and these should be investigated further.

In conclusion, we confirmed that age, sex, Ce_LOC_, and both total fentanyl and propofol consumption amounts were independent factors that predicted Ce _ROC_ during propofol infusion under TCI with the Schnider model. Our results are applicable to clinical practice. Patients can be expected to awaken quickly upon completion of the procedure and discontinuation of drug administration. However, further study is needed to investigate the impact on OR turnover rate.
